# Evaluation of clinical significance of TP53, BCL-2, BAX and MEK1 expression in 229 ovarian carcinomas treated with platinum-based regimen

**DOI:** 10.1038/sj.bjc.6600789

**Published:** 2003-03-18

**Authors:** J Kupryjańczyk, T Szymańska, R Mądry, A Timorek, J Stelmachów, G Karpińska, A Rembiszewska, I Ziółkowska, E Kraszewska, J Dębniak, J Emerich, M Ułańska, A Płużańska, M Jędryka, M Goluda, A Chudecka-Głaz, I Rzepka-Górska, M Klimek, K Urbański, J Bręborowicz, J Zieliński, J Markowska

**Affiliations:** 1 Department of Molecular Pathology, Institute of Oncology, Roentgena 5, 02-781 Warsaw, Poland; 2 Chair of Oncology, University of Medical Sciences, Poznań, Poland; 3 Department of Obstetrics and Gynecology, Bródnowski Hospital and II-nd Faculty of Medicine, Medical University, Warsaw, Poland; 4 Department of Pathology, Bródnowski Hospital and II-nd Faculty of Medicine, Medical University, Warsaw, Poland; 5 Department of Gynecologic Oncology, Institute of Oncology, Roentgena 5, 02-781 Warsaw, Poland; 6 Department of Biostatistics, Institute of Oncology, Roentgena 5, 02-781 Warsaw, Poland; 7 II-nd Gynecological Department, Medical University, Gdańsk, Poland; 8 Department of Chemotherapy, Medical University, Łodź, Poland; 9 II-nd Department of Gynecology, Medical Academy, Wrocław, Poland; 10 Department of Gynecological Surgery and Oncology of Adults and Adolescent, Pomeranian Academy of Medicine, Szczecin, Poland; 11 Department of Gynecologic Oncology, Institute of Oncology, Kraków, Poland; 12 Department of Pathology, University of Medical Sciences, Poznań, Poland

**Keywords:** TP53, MEK, BCL-2, BAX, ovarian cancer

## Abstract

In cell line studies, BCL-2, BAX, as well as novel MEK1 protein levels have strong influence on ovarian cancer response to cisplatin-based chemotherapy. However, such associations have not been demonstrated clinically. We evaluated prognostic/predictive significance of these proteins with regard to TP53 status. Immunohistochemical analysis was performed on 229 ovarian carcinomas FIGO stage IIB–IV treated with platinum-based chemotherapy; the results were analysed by the Cox and logistic regression models. Clinical parameters (residual tumour size, patient age, FIGO stage) were the only indicators of overall survival (OS) and the strongest predictors of complete remission (CR). On the other hand, BAX expression was the strongest (*P*=0.005) or the only (in FIGO IIIC, *P*=0.02) prognostic indicator of disease-free survival (DFS) in the TP53(+) group. TP53(+) and TP53(−) ovarian carcinomas differed in clinical and molecular prognostic and predictive factors. Another novel finding is that CR was negatively influenced by high BAX expression in all patients group (*P*=0.047) and by BCL2 expression in the TP53(−) group (*P*=0.05). High MEK1 expression was associated with endometrioid and clear cell carcinomas (*P*=0.049); its loss was found with advancing FIGO stage (*P*=0.002). Our results suggest that binomial TP53 status divides ovarian carcinomas into two biologically distinct groups. BAX expression is an important factor of DFS in the TP53(+) group. BCL-2 and BAX, but not MEK1 expressions have predictive value in ovarian cancer patients treated with platinum-based chemotherapy.

Cisplatin is the most commonly used chemotherapeutic compound in ovarian cancer patients and resistance to it is a major clinical problem in this condition (Christian and Trimble, 1994). Apoptosis is a predominant mechanism of tumour cell loss during chemotherapy, and its inefficiency may be an important cause of chemoresistance. Cisplatin induces apoptosis, which in a majority of ovarian carcinoma cell lines is TP53-dependent (Jones *et al*, 1998; Shimada *et al*, 2000). Impaired TP53 protein function, most frequently reflecting *TP53* gene mutation contributes to resistance to cisplatin in ovarian carcinoma cell lines (Herod *et al*, 1996; Jones *et al*, 1998; Shimada *et al*, 2000), and the same could be expected clinically. Despite high frequency of *TP53* gene mutations in ovarian carcinomas (Kupryjańczyk *et al*, 1993,^2000^; Casey *et al*, 1996; DiCioccio *et al*, 1998; Wen *et al*, 1999), studies on TP53 status and tumour response to cisplatin-based chemotherapy have not so far given equivocal results (Righetti *et al*, 1996; de Feudis *et al*, 1997; Baekelandt *et al*, 1999; Eisenhauer *et al*, 1999; Fallows *et al*, 2001; Reles *et al*, 2001).

TP53 cooperates with apoptosis-regulating proteins in tumour response to cisplatin-based chemotherapy. *BCL-2* expression (an apoptosis inhibitor) is downregulated, while *BAX* expression (an apoptosis promoter) is upregulated by TP53 protein (Miyashita *et al*, 1994; Miyashita and Reed, 1995). It has also been shown that cisplatin-induced apoptosis is associated with wild-type TP53/BAX complex formation (Raffo *et al*, 2000). Cell line studies show that TP53-regulated protein levels may differ constitutionally and/or after cisplatin administration depending on the functional TP53 status (Herod *et al*, 1996; Jones *et al*, 1998). Thus, in multivariate analyses of prognostic and/or predictive factors, evaluating tumours together with functional and dysfunctional TP53 protein may possibly mask the biological significance of proteins regulated by or interacting with wild-type TP53 but not with mutant TP53.

Studies on ovarian carcinoma cell lines have revealed an association between high BCL-2 levels and resistance to cisplatin (Eliopoulos *et al*, 1995; Herod *et al*, 1996; Jones *et al*, 1998), however, contradictory results have been published, too (Beale *et al*, 2000); BAX expression either enhanced or did not influence ovarian carcinoma cell lines sensitivity to cisplatin (Jones *et al*, 1998; Beale *et al*, 2000). Despite these findings, significance of BCL-2 or BAX expression in ovarian cancer response to cisplatin-based chemotherapy has not been confirmed by clinical studies.

MEK is a relatively recently described apoptosis inhibitor. MEK (MEK1/MEK2 isoforms) is a MAP kinase kinase that plays a role in signal transduction from growth factors in a receptor tyrosine kinase – RAS–RAF–MEK–ERK cascade (Garrett and Workman, 1999). This cascade transmits both mitogen and antiapoptotic signals. It has been shown that MEK may stimulate antiapoptotic BCL-2, BCL-XL and MCL-1 proteins (Boucher *et al*, 2000), as well as inactivate proapoptotic protein BAD (Scheid and Duronio, 1998). Cell line studies on the role of MEK kinase in tumour response to cisplatin-based chemotherapy are controversial: in some studies MEK1 inhibited (Hong *et al*, 1999), in others activated TP53-dependent apoptosis after cisplatin administration; in the latter case the apoptosis was completely blocked by MEK1 inhibitors (Ryan *et al*, 2000; Wang *et al*, 2000).

Analyses of TP53 and/or apoptosis proteins in large groups of ovarian carcinomas are rare (Hartmann *et al*, 1994; Eltabbakh *et al*, 1997; Marx *et al*, 1997; Baekelandt *et al*, 1999,^2000^; Ferrandina *et al*, 1999). To our knowledge, MEK1 expression and its clinical significance have not been evaluated yet in ovarian carcinomas. In this study, we present an analysis of the clinical significance of TP53 accumulation and expression of proteins interacting with TP53 in tumour response to cisplatin, that is BCL-2, BAX and MEK1. We also present an alternative approach to the analysis of prognostic and predictive factors in ovarian carcinomas by eliminating variability of the TP53 status.

## MATERIALS AND METHODS

### Patients and tumours

The study was performed on archival ovarian carcinomas from 229 patients treated in eight gynaecologic oncology centres in Poland. The material was carefully selected out of 548 cases submitted to meet the following criteria: no chemotherapy before staging laparotomy, adequate staging procedure, FIGO stage IIB to IV, standard CP (cisplatin–cyclophosphamide or carboplatin–cyclophosphamide) or CAP chemotherapy (CP with addition of doxorubicin), and tumour tissue from the first laparotomy available. Medical records were critically reviewed by at least two clinicians. Patients ranged in age 24–77 years (median 53.2). Tumours were staged according to the criteria of the International Federation of Gynaecologists and Obstetricians (Creasman, 1989) (Table 1

**Table 1:** Tumour characteristics in the TP53-negative and TP53-positive group

	**TP53(−) *N*=94**	**TP53(+) *N*=135**	***P*-value^a^**
*Age*			
Range	24–76	25–77	0.57
mean (s.d.)	52.4 (11.1)	53.7 (9.9)	
			
*FIGO stage*			
IIB	4 (4%)	6 (4%)	
IIC	2 (2%)	5 (4%)	
IIIA	4 (4%)	4 (3%)	0.8
IIIB	19 (20%)	26 (19%)	
IIIC	51 (54%)	78 (58%)	
IV	14 (15%)	16 (12%)	
			
*Residual tumour*			
0–2 cm	44 (47%)	65 (48%)	0.92
>2 cm	50 (53%)	70 (52%)	
			
*Histological type*			
Serous	63 (67%)	114 (84%)	
Endometrioid	9 (10%)	5 (4%)	0.001
Clear cell	12 (13%)	0	
Undifferentiated	5 (5%)	8 (7%)	
Other	5 (5%)	7 (5%)	
			
*Histological grade*			
G2	19 (20%)	11 (8%)	
G3	55 (59%)	87 (64%)	0.032
G4	20 (21%)	37 (27%)	
			
*Chemotherapy*			
CP	70 (75%)	95 (70%)	
CAP	24 (25%)	40 (30%)	
			
*Response to chemotherapy*			
Complete remission	46 (49%)	74 (55%)	
Partial remission	11 (12%)	24 (18%)	
No change	4 (4%)	4 (3%)	
Progression	33 (35%)	33 (24%)	
			
Platinum resistant	53 (56%)	77 (57%)	
Platinum sensitive	41 (44%)	58 (43%)	
			
Recurrence rate in a CR group	34/46 (74%)	58/74 (78%)	
			
*Outcome*			
NED	12 (13%)	23 (17%)	
AWD	9 (10%)	17 (13%)	
DOD	70 (75%)	94 (70%)	
DOC	3 (3%)	1 (1%)	

CP=cyclophosphamide and cisplatin, CAP=CP plus doxorubicin, CR=complete remission, NED=no evidence of disease, AWD=alive with disease, DOD=died of disease, DOC=died of other causes

a *χ*^2^ test.

). All tumours were uniformly reviewed histopathologically, classified according to the criteria of the World Health Organization (Russell, 1994) and graded in a four-grade scale according to the criteria given by (Barber *et al*, 1975) (Table 1).

Follow-up time was stated on the basis of patient's date of death or the last information present in medical records. Follow-up time ranged 1.44–146.7 months (median 24.7) and 168 patients have died (73%). Follow-up time for the group of still alive patients ranged 7–146.7 months (median 41). Patients outcome is shown in Table 1.

### Evaluation of clinical response to chemotherapy

Response to chemotherapy was evaluated retrospectively according to the World Health Organization response evaluation criteria (Miller *et al*, 1981). The evaluation was based on data from medical records describing patient's clinical condition and CA125 levels in 3–4 week intervals. Complete remission (CR) was defined as disappearance of all clinical and biochemical symptoms of ovarian cancer evaluated after completion of first-line chemotherapy and confirmed at 4 weeks. Within the CR group we have defined a platinum-sensitive (PS) group (disease-free survival (DFS) longer than 6 months, 99 patients). The other tumours (partial remission – PR, progression – P, no change – NC), as well as the CR group with DFS shorter than 6 months were described as resistant to cisplatin (Christian and Trimble, 1994) (Table 1).

### Immunohistochemical analysis

All immunohistochemical stainings were performed on paraffin-embedded material after heat-induced epitope retrieval (HIER). Owing to the multicenter origin of paraffin blocks, all tumours were checked as to the immunoreactivity by staining for vimentin. Intratumoural inflammatory infiltrate stained for BCL-2 and BAX was a control for tissue immunoreactivity, too. We used PAb1801 monoclonal antibody (1 : 500, Sigma-Genosys, Cambridge, UK) for TP53 protein, anti-MEK1 (clone H-8) and anti-BAX (clone B-9) monoclonal antibodies (both 1 : 80, Santa Cruz Biotechnology Inc, Santa Cruz, USA), anti-BCL-2 monoclonal antibody (1 : 80, clone 124, Dako, Glostrup, Denmark) and antivimentin monoclonal antibody (1 : 50, clone V9, Immunotech, Marseille, France). Deparaffinised sections were boiled in a citrate buffer (pH 6.0) at 700 W in a microwave: 2 × 5 min for TP53 and vimentin, 3 × 5 min for BCL-2 and 6 × 5 min for BAX; for MEK1 detection, the sections were boiled in the same buffer for 30 s at 120°C and 15 psi in an autoclave. Nonspecific tissue and endogenous peroxidase reactivity were blocked with 10% BSA and 3% H_2_O_2_, respectively. Tissue sections were incubated with primary antibodies for 1 h at room temperature (antivimentin, anti-BCL-2 and anti-TP53) or overnight at 4^o^C (the other). Biotinylated goat anti-mouse IgG (1 : 1500, cat. no. 816), peroxidase-conjugated streptavidin (1 : 500, cat. no. 309) (both from Immunotech, Marseille, France) and DAB were used as a detection system. Ovarian carcinomas with and without *TP53* gene mutation were controls for TP53. Intratumoural lymphocytes and plasma cells, as well as a tonsil tissue were controls for BCL-2 and/or BAX. Normal salpingeal mucosa from three cases studied served as a positive control for MEK1; anti-WAF1 monoclonal antibody from the same company as anti-MEK1, detecting a nuclear antigen served as an isotype-matched antibody for anti-MEK1. Normal mouse IgG of the same subclasses and concentrations as the primary antibodies served as negative controls, too.

Semiquantitative evaluation of immunohistochemical stainings was performed independently by two pathologists (JK, TSZ). TP53 protein accumulation was described as present (more than 10% of positive cells) or absent. BAX and MEK1 expressions were described as: (1) negative, trace or weak (further called low), (2) moderate and (3) strong (both further called high). BCL-2 expression was described as: (1) negative or focal, and (2) positive.

### Statistical analysis

Probability of survival and DFS were estimated using the Kaplan–Meier method (Kaplan and Meier, 1958). Overall and DFS time analyses were performed with multivariate Cox's proportional hazards models (Cox, 1972); tumour response to chemotherapy (probability of CR, probability of PS response) was evaluated with the multivariate logistic regression model. Important factors were selected using backward selection technique, where factors not significant at 0.1 were drawn one by one out of the model. The analysis was performed in all ovarian carcinomas, and separately in the TP53(−) and TP53(+) subgroups. To eliminate an influence of stage parameter, we evaluated separately FIGO IIIC group, too.

Associations between protein expressions and histological type, grade, FIGO stage and residual tumour size were studied by *χ*^2^ test (Mehta and Patel, 1983). All tests were two-sided and the level of significance was set at 5%. All calculations were done using the STATA 6.0 program.

## RESULTS

### TP53, BCL-2, BAX and MEK1 associations

TP53 protein accumulation was observed in malignant cells only, while the other proteins, that is BCL-2, BAX and MEK1 were expressed by normal cells or tissues, as well. In particular, MEK1 protein was expressed by mucosa of the uterine tube and inconstantly by fibroblasts and mesothelium. BCL-2 showed heterogeneous, cytoplasmic staining; BAX and MEK1 showed cytoplasmic rather homogeneous staining.

**Figure 1:**
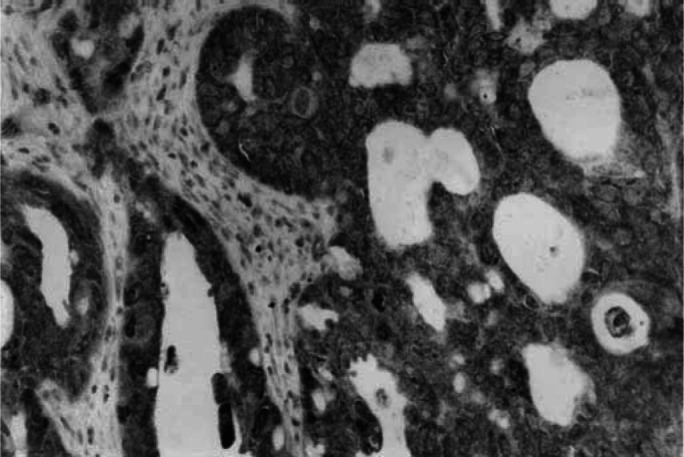
Strong cytoplasmic MEK1 expression in an endometrioid carcinoma of the ovary (haematoxylin counterstain, × 250).

TP53 protein nuclear accumulation was present in 135 (59%) tumours. BCL-2 was negative in majority of cases (*N*=156; 68%); it was positive in 73 cases (32%). BAX expression was low in 85 (37%) cases; it was moderate in 57 (25%) and strong in 87 (38%) cases. MEK1 expression was low in 52 tumours (23%); it was moderate in 98 (43%) and strong in 79 (34%) tumours (Figure 1

). BCL-2, BAX and MEK1 protein expressions were not associated with each other, neither with TP53 protein accumulation.

Associations of protein expressions with histological tumour types are shown in Table 2

**Table 2:** Associations of protein expressions with histological tumour types

	**Serous (%)**	**Undifferentiated and other (%)**	**Endometrioid and clear cell (%)**	***P*-value**
TP53 positive	66	61	30	0.001
BCL-2 positive	27	28	62	0.003
BAX highly positive	59	85	65	0.043
MEK1 highly positive	75	77	96	0.049

. Poor tumour differentiation/undifferentiation was associated with TP53 protein accumulation (*P*=0.032) and high BAX positivity (*P*=0.018). BCL-2 and MEK1 expressions did not correlate with tumour grade.

Loss of intensity of MEK1 expression was apparent concomitantly with advancing FIGO stage (100% high positivity for IIB and IIC, 91% for IIIA and IIIB, 70% for IIIC and IV) (*P*=0.002). Other protein expressions did not correlate with FIGO stage.

### Overall survival analysis

Overall survival was associated with clinical parameters and not with any protein expression studied. Overall survival in the whole group was influenced by patient age, FIGO stage and residual tumour (RT) size (Table 3

**Table 3:** Overall survival (risk of death) in the whole group of ovarian carcinomas, and in the TP53(+) and TP53(−) group (Cox's proportional hazards model)

	**TP53(−) and (+) *N*=229, 168 deaths**			**TP53(−) *N*=94, 73 deaths**			**TP53(+) *N*=135, 95 deaths**		
	**RR**	**95% CI for RR**	***P***	**RR**	**95% CI for RR**	***P***	**RR**	**95% CI for RR**	***P***
Age									
⩾52 *vs* <52	1.9	[1.4, 2.6]	<0.001			0.11	1.9	[1.3, 3.0]	0.002
FIGO			0.011			0.045			0.17
IIB, IIC *vs* IV	0.21	[0.08, 0.55]	0.001	0.09	[0.01, 0.68]	0.020			
IIIA, IIIB *vs* IV	0.55	[0.31, 0.97]	0.04	0.47	[0.23, 0.98]	0.045			
IIIC *vs* IV			0.23			0.28			
Residual tumour									
>2 cm *vs* 0–2 cm	1.8	[1.2, 2.6]	0.004			0.28	2.4	[1.6, 3.8]	0.0001
Chemotherapy									
CAP *vs* CP	1.4	[0.98, 1.9]	0.07	1.6	[0.93, 2.7]	0.09			0.51
Histological type			0.06			0.37			0.79
Endo/clear *vs* ser	1.7	[1.0, 2.7]	0.04						
Undiff/other *vs* ser			0.16						

Endo/clear=endometrioid or clear cell; ser=serous; undiff/other=undifferentiated or other types.

). In addition, risk of death was higher in cases with endometrioid and clear cell-type carcinoma of the ovary when compared with the serous ones.

Overall survival analysis in the TP53(+) and TP53(−) subgroups revealed two prognostically different classes of ovarian carcinomas (Table 3; only variables showing associations in at least one analysis are shown in the tables). Overall survival was negatively associated with higher patient age and larger RT in the TP53(+) group, but not in the TP53(−) group. FIGO stage influenced OS in the TP53(−) group, but not in the TP53(+) group.

Chemotherapy with the addition of doxorubicin compared with CP regimen showed a tendency to increased risk of death in all patients group (*P*=0.07) (Table 3). It turned out in further analysis that FIGO stage IIIC TP53(−) group showed higher probability of death when treated with CAP chemotherapy (Table 4

**Table 4:** Overall survival (risk of death) in FIGO IIIC ovarian carcinomas, and in the group IIIC divided according to TP53 status (Cox's proportional hazards model)

	**TP53(−) and (+) *N*=129, 101 deaths**			**TP53(−) *N*=51, 41 deaths**			**TP53(+) *N*=78, 60 deaths**		
	**RR**	**95% CI for RR**	***P***	**RR**	**95% CI for RR**	***P***	**RR**	**95% CI for RR**	***P***
Age									
⩾52 *vs* <52	3.1	[2.0, 4.7]	<0.001	3.1	[1.6, 5.9]	<0.001	2.9	[1.7, 5.0]	<0.001
Chemotherapy									
CAP *vs* CP			0.096	2.2	[1.0, 4.6]	0.04			0.46

). The only factor that consequently influenced OS in the FIGO IIIC group was patient age Table 4).

### Complete remission and platinum sensitivity

Complete remission (CR) was achieved in 120 patients (52%), while PS response (CR with DFS longer than 6 months) was achieved in 99 of 229 patients (43%). Clinical parameters were the strongest predictors of CR (Table 5

**Table 5:** Probability of CR in the whole group of ovarian carcinomas, and in the TP53(+) and TP53(−) group (logistic regression model)

	**TP53(−) and (+) *N*=229, 120 CR**			**TP53(−) *N*=94, 46 CR**			**TP53(+) *N*=135, 74 CR**		
	**OR**	**95% CI for OR**	**P**	**OR**	**95% CI for OR**	**P**	**OR**	**95% CI for OR**	***P***
*Age*									
⩾52 *vs* <52	0.49	[0.27, 0.89]	0.019	0.36	[0.14, 0.88]	0.026			0.28
*Residual tumour*									
>2 cm *vs* 0–2 cm	0.16	[0.08, 0.33]	<0.001	0.27	[0.11, 0.68]	0.005	0.10	[0.04, 0.22]	<0.001
*BAX*									
High (+) *vs* low (+)	0.53	[0.29, 0.99]	0.047			0.52			0.13
*BCL-2*									
(+) *vs* (−)			0.14	0.4	[0.16, 1.01]	0.05			0.99

) and the only predictors of PS response. Nevertheless, CR was associated with the apoptosis proteins, too.

Complete remission status was associated with small RT size in all three groups analysed (Table 5). It was also associated with lower patient age in the whole group and in the TP53(−) group. Complete remission status was associated with low BAX staining in the whole group, and with negative BCL-2 staining in the TP53(−) group. The only factor that influenced probability of CR in the TP53(+) group was RT size (Table 3).

Platinum sensitivity showed associations with small RT at the same *P* level as CR; PS was also associated with FIGO stage IIB or IIC in the whole group (*P*=0.02) and in the TP53(+) group (*P*=0.05), as well as with patient age in the whole group only (*P*=0.017). Low BAX expression showed a tendency to increase probability of PS in the TP53(−) group, but it was at the border of significance (*P*=0.07). Other factors did not influence response to chemotherapy.

### Disease-free survival analysis

In the whole group of patients with CR (*N*=120, 92 with recurrences), DFS was influenced by FIGO stage only (*P*=0.0005).

In the TP53(+) group (74 with CR, 58 with recurrences) BAX expression was the strongest or the only (in FIGO IIIC, *P*=0.02) prognostic indicator of DFS. In this group, DFS was longer with lower FIGO stage (*P*=0.01) and with high BAX expression (*P*=0.005, RR=0.45, 95% CI for RR: [0.25, 0.78]). This tendency has been confirmed in the TP53(+) FIGO IIIC group (39 with CR, 32 with recurrences), that is after elimination of clinical stage parameter: strong (not generally high) BAX expression had positive influence on DFS (*P*=0.02, RR=0.39, 95% CI for RR: [0.17, 0.88]). Strong BAX expression in the TP53(+) group was the only parameter that influenced DFS in FIGO IIIC group.

In the TP53(−) group (46 with CR, 34 with recurrences) DFS was longer with lower residual tumour, at the border of significance (*P*=0.06). The other factors included into the analysis did not influence DFS time.

## DISCUSSION

In cell line studies, BCL-2, BAX, as well as novel MEK1 protein levels have strong influence on ovarian cancer response to cisplatin-based chemotherapy. With a single exception related to BCL-2 expression (Mano *et al*, 1999), such associations have not been demonstrated clinically. To our knowledge, this is the first evidence of influence of BAX and BCL-2 expression on CR in ovarian cancer patients, confirmed by multivariate analysis. BAX expression, in contrast to its apoptosis promoter function and similarly to BCL-2 expression had negative impact on CR. Similar discrepancy between biological function of apoptosis proteins and their clinical significance has been previously observed by Marx *et al* (1997) who found a bad prognostic significance of BAX expression, and a good one of BCL-2 expression.

In the current study, negative impact of BCL-2 expression on CR was demonstrated in the TP53-negative carcinomas only, confirming our hypothesis that TP53-positive and TP53-negative ovarian carcinomas differ in clinical significance of apoptosis proteins interacting with TP53 in tumour response to cisplatin-based chemotherapy. In subgroups related to the TP53 status, BCL-2 and BAX demonstrated an influence on CR or DFS, which was not seen in all tumours. Among other studies evaluating predictive significance of BCL-2 (Herod *et al*, 1996; Mano *et al*, 1999; Schuyer *et al*, 2001), including two published by Baekelandt *et al*, (1999,2000) on a comparable number of ovarian carcinomas, only Mano *et al* (1999) noticed higher frequency of CR in patients with BCL-2 negative tumours.

In regard to BAX expression, a few studies have addressed the issue of its clinical significance in advanced stage ovarian carcinomas; however, associations with CR or DFS have not been observed (Marx *et al*, 1997; Baekelandt *et al*, 2000; Sengupta *et al*, 2000; Schuyer *et al*, 2001). Recently, Schuyer *et al* (2001) found an association of BAX expression with progression-free survival.

Interestingly, TP53-positive and TP53-negative carcinomas demonstrated striking differences not only in relation to immunohistochemical markers, but also to clinical parameters. After division of ovarian carcinomas into TP53-negative and TP53-positive, prognostic significance of patient age, FIGO stage, residual tumour size and histological tumour type either disappeared or became weaker or stronger, depending on the group. As a factor of OS, FIGO stage appeared to be an alternative to patient age. This has been supported by the analysis of FIGO IIIC group only, in which patient age was a constant prognostic factor irrespective of TP53 status. Apparently, the differing results for the TP53-negative and TP53-positive carcinomas were not influenced by differences in clinicopathological characteristics, since both groups were very similar. Nevertheless, the tendencies revealed by subgroup analysis should be confirmed on larger population.

Despite the novel findings related to TP53 and apoptosis proteins, clinical parameters were the only factors of OS, the strongest predictors of CR and the only predictors of platinum sensitivity. In particular, residual tumour size was a constant and important parameter influencing CR. The strong impact of clinical parameters on clinical end points in our analysis is in agreement with other studies (Makar *et al*, 1995; Partridge *et al*, 1996; Eisenhauer *et al*, 1999). Usually, immunohistochemical markers present lower associations with clinical end points than clinical parameters (Diebold *et al*, 1996; Herod *et al*, 1996; Baekelandt *et al*, 1999,^2000^; Mano *et al*, 1999; Geisler *et al*, 2000; Reles *et al*, 2001; Schuyer *et al*, 2001). In our analysis, the only exception was the positive influence of high BAX expression on DFS in patients with TP53-positive ovarian carcinomas – it was stronger than that of the FIGO stage. After elimination of stage factor (TP53-positive FIGO IIIC group only) BAX expression was the only parameter that influenced DFS.

Evaluation of clinical significance of MEK1 and TP53 expression has generally brought negative results, confirming that observations from cell line studies cannot be directly referred to clinical situations. As far as TP53 is concerned, in the literature there are few studies on clinical significance of TP53 protein accumulation in large groups of advanced stage ovarian carcinomas (Hartmann *et al*, 1994; Eltabbakh *et al*, 1997; Baekelandt *et al*, 1999; Ferrandina *et al*, 1999; Shahin *et al*, 2000; Reles *et al*, 2001). Two of three of those research groups who related TP53 protein accumulation to tumour response to platinum-based chemotherapy have found better response in TP53-negative tumours (Ferrandina *et al*, 1999; Reles *et al*, 2001). Baeklandt *et al* (1999) have shown a prognostic but not predictive significance of TP53 protein accumulation, while other authors did not find any clinical significance of the TP53 protein (Hartmann *et al*, 1994; Eltabbakh *et al*, 1997; Shahin *et al*, 2000). Similarly, in our study TP53 expression by itself did not show any clinical significance. Interestingly though, our results suggest that impaired TP53 protein function may level or enhance prognostic or predictive significance of other factors. Platinum compounds and taxanes that are currently applied as a standard treatment in ovarian cancer patients have different molecular targets. There is a pilot study showing that tumours with *TP53* gene mutations show better response to paclitaxel than tumours with wt TP53 (Lavarino *et al*, 2000); the reverse is observed by some authors in relation to cisplatin (as above). It seems that separate evaluation of TP53-positive and TP53-negative subgroups may help to identify molecular profiles of tumours, which will show differential response to different chemotherapeutic regimens.
